# Psychological stress catalyses free radical–mediated activation of coagulation in humans

**DOI:** 10.1113/JP290576

**Published:** 2026-04-25

**Authors:** Lewis Fall, Benjamin S. Stacey, Elsie Swain, Ebony Samuel, Meghan Robinson, Danny W. Walmsley, Christopher J. Marley, Thomas S. Owens, Adnan Haq, Leon G. Yandle, Kevin Cox, Damian M. Bailey

**Affiliations:** ^1^ Neurovascular Research Laboratory University of South Wales Pontypridd UK; ^2^ Faculty of Computing, Engineering and Science University of South Wales Pontypridd UK; ^3^ Faculty of Life Sciences and Education University of South Wales Pontypridd UK; ^4^ Swansea University Medical School Swansea UK; ^5^ Bexorg, Inc. New Haven Connecticut USA

**Keywords:** haemostasis, oxidative stress, stress

## Abstract

**Abstract:**

Psychological stress is a recognised, yet mechanistically unresolved, risk factor for cardiovascular disease (CVD) partly through its association with a hypercoagulable state. Free radical–mediated oxidative stress has been proposed as a key upstream driver of this haemostatic imbalance. In this randomised cross‐over study we investigated whether acute psychological stress promotes systemic radical formation and prothrombotic alterations in clot microstructure in eight healthy males. The Trier Social Stress Test was used to induce psychological stress. Antecubital venous blood was collected to measure the ascorbate free radical (A^•−^, electron paramagnetic resonance spectroscopy) and clot microstructure (D_f_, Fourier transform rheology), alongside standard coagulometry. Compared with the control condition (quiet sitting), psychological stress increased A^•−^ (*P* = 0.042) and D_f_ (*P* = 0.008), the latter reflecting larger, denser and more fibrin‐rich networks. We also observed selective shortening of activated partial thromboplastin time (aPTT) (*P* = 0.018), indicating activation of the intrinsic coagulation pathway. This study provides the first *in vivo* evidence that acute psychological stress triggers systemic free radical formation and drives prothrombotic remodelling of clot architecture. These findings identify oxidative stress as a mechanistic link between psychological stress and CVD risk and highlight it as a compelling target for prevention and therapy.

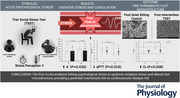

**Key points:**

- Although psychological stress is a recognised risk factor for cardiovascular disease (CVD), its mechanistic association with a hypercoagulable state remains unresolved.
- Acute psychological stress, induced by the Trier Social Stress Test (TSST), significantly increased systemic free radical formation, as measured by elevated levels of ascorbate free radical (A^•−^) via electron paramagnetic resonance (EPR) spectroscopy, and was associated with the formation of larger, denser, more fibrin‐rich clots confirmed by increased fractal dimension (D_f_).
- The concurrent increase in A^•−^ and D_f_ suggests that free radical–mediated oxidative stress is an upstream driver of psychological stress‐induced activation of haemostasis, specifically altering clot quality.
- The TSST selectively shortened activated partial thromboplastin time (aPTT), indicating activation of the intrinsic/contact coagulation pathway. There were no changes in prothrombin time (PT) or D‐dimer, suggesting haemostatic activation occurred without engaging fibrinolysis.
- The findings demonstrate that even a brief episode of emotional stress can increase thrombotic potential in healthy individuals and identify oxidative stress as a key mechanistic link and a potential therapeutic target for treating stress‐related CVD.

## Graphical abstract

**Abstract figure legend** This study provides the first *in vivo* evidence that acute psychological stress triggers systemic free radical formation and prothrombotic remodelling of blood clot microstructure. By inducing stress in healthy participants, we observed a significant increase in oxidative stress (A^•−^), a shortening of the intrinsic pathway of coagulation (activated partial thromboplastin time (aPTT)) and the formation of denser, more fibrin‐rich clot networks (D_f_). These findings identify oxidative stress as a critical mechanistic link between psychological stress and cardiovascular disease, highlighting it as a potential target for preventative therapies. Created in https://BioRender.com.

## Introduction

Cardiovascular disease (CVD) is one of the leading causes of death and disability worldwide (Roth et al., 2020), and is responsible for one‐third of global mortality (Lindstrom et al., 2022). Traditional risk factors of CVD go only part‐way to explaining overall CVD risk (Greenland et al., 2003), and behavioural and emotional triggers have long been suspected as playing a vital, precipitating role in atherothrombotic events subsequent to (mal)activation of the haemostatic system (Strike & Steptoe, 2005).

Indeed it is well known that the *in vivo* balance of the haemostatic system can be disrupted by various forms of emotional or psychological stress, resulting in a hypercoagulable state (Austin et al., 2011, 2012; Cannon, 1929; Neuenfeldt et al., 2021; Thrall et al., 2007; Von Känel et al., 2001), although the underlying mechanisms remain poorly understood. Different mechanisms have been postulated, including catecholamine‐mediated inflammation (Borissoff et al., 2011), increased shear stress (Austin et al., 2013) and haemoconcentration (Austin et al., 2012), direct platelet activation (Von Känel & Dimsdale, 2000) and broader stress‐induced inflammatory‐coagulation cross‐talk (von Känel et al., 2005). However the stress‐haemoconcentration hypothesis remains disputed (Fall & Bailey, 2013).

Emerging evidence from our laboratory supports the concept that free radical formation represents an important initiating biochemical factor in the dysregulation of the haemostatic system (Fall et al., 2015, 2018, 2023). The literature further suggests that psychological stress, via activation of the hypothalamic‐pituitary‐adrenal (HPA) axis (Herman & Tasker, 2016), enhances mitochondrial oxidation, membrane potential and calcium‐holding capacity (Du, Wang et al., 2009), ultimately promoting superoxide anion generation and downstream oxidative stress (Spiers et al., 2014).

To investigate this we conducted a randomised controlled cross‐over trial to determine whether a single acute bout of psychological stress alters systemic free radical formation and haemostatic function. Systemic free radicals were quantified using electron paramagnetic resonance (EPR) spectroscopy, whereas Fourier transform haemorheology was employed to characterise, for the first time, the microstructure of human incipient blood clots under these experimental conditions. We hypothesised that acute psychological stress would increase systemic free radical accumulation and induce corresponding alterations in incipient clot microstructure.

## Methods

### Ethical approval and participants

The study was approved by the University of South Wales Ethics Committee (26/2/24/LF/1). Eight healthy men aged 21 (mean) ± (SD) 3 years were recruited (see the ‘Statistical Analysis’ section for power calculations). Participants were free of CVD, not taking supplements and instructed to refrain from physical activity, caffeine and alcohol; follow a low‐nitrate/low‐nitrite diet for 24 h (Bailey et al., 2017); and attend the laboratory after a 12‐h overnight fast. All procedures were carried out in accordance with the *Declaration of Helsinki* of the World Medical Association with the exception of being registered in a database (Williams, 2008). Written informed consent was obtained from all participants.

### Design

A randomised controlled cross‐over design was employed. Participants completed either a control condition (quiet sitting) or the Trier Social Stress Test (TSST), with the alternate condition performed 1 week later. Venous blood samples were collected immediately before and after each intervention for all outcome measures.

### Intervention

The TSST, the gold standard for assessing acute psychosocial stress (Allen et al., 2017), consisted of three consecutive 5‐min phases: (1) anticipatory stress, during which participants prepared a speech before a judge and camera, with notes unexpectedly removed before speaking; (2) presentation, in which the judge maintained a neutral expression throughout; and (3) mental arithmetic, requiring backward counting from 2003 in steps of 17, restarting after any error. Sessions ended with recovery and debriefing.

### Blood sampling

An 18‐gauge cannula (Venflon IV cannula, Becton‐Dickinson, Stockholm, Sweden) connected to a three‐way sterile stopcock (Connecta plus 3, Ohmeda, Sweden) was inserted into a prominent antecubital vein. Whole blood was collected without stasis into a sterile syringe for immediate analysis of haemorheological markers of blood clot microstructure. We then obtained separate samples for direct detection of traditional markers of coagulation activation, absolute blood viscosity and ascorbate free radical (A^•−^) using the vacutainer method (Becton, Dickinson and Company, Oxford, UK). Vacutainers were centrifuged at 600 *g* (4°C) for 10 min, and plasma supernatant (sodium citrate or K‐EDTA) was decanted into cryogenic vials (Nalgene Labware, Thermo Fisher Scientific Inc., Waltham, MA, USA) and either gently frozen to −80°C (citrated plasma) or immediately snap frozen in liquid nitrogen (K‐EDTA). Plasma samples were thawed at 37°C for 5 min prior to batch analysis.

### Biomarkers

***Free radicals***: EPR was employed to directly measure A^•−^ in K‐EDTA plasma as a global biomarker of free radical formation (Bailey et al., 2018). Exactly 1 mL of K‐EDTA plasma was injected into a high‐sensitivity multiple‐bore sample cell (AquaX, Bruker Daltonics, Billerica, MA, BD, USA) housed within a TM110 cavity of an EPR spectrometer (BD, USA) operating at X‐band (9.87 GHz). Samples were analysed using a modulation frequency of 100 kHz, modulation amplitude of 0.65 gauss (G), microwave power of 10 mW, receiver gain of 2×10^5^ AU, time constant of 41 ms, magnetic field centre of 3477 G and scan width of ±50 G for three incremental scans. After identical baseline correction and filtering, each of the spectra was subject to double integration using graphical analysis software (OriginPro, version 8.5, OriginLab, MA, USA). Both intra‐ and inter‐assay coefficient of variations for all measured metabolites were less than 10% (Bailey et al., 2018).

***Clot microstructure***: Blood clot microstructure was obtained via Fourier transform rheology. Exactly 7 mL of unadulterated whole blood was immediately injected into a double‐walled concentric rheometer (Discovery Hybrid‐2, TA Instruments, DE, USA) for analysis of D_f_ at 37°C according to established methods (Evans et al., 2010). Briefly blood was subject to a constant torque of 10.5 µNm at 2, 0.93, 0.43 and 0.2 Hz rotational oscillation. The phase angle (δ) of the insipient clot was determined by storage modulus (*G*′)/loss modulus (*G*″) frequency harmony. Time for blood to gel (*T*_GP_) and dynamic viscosity (DV) were recorded from this point, and the corresponding D_f_ of the insipient clot was calculated according to the established relationship:

$$\frac{\left(D+2\right)\left(2\theta -D\right)}{2\left(\theta -D\right)}$$

where *D* is the space dimension (constant of 3 arbitrary units) and the exponent (δ) was calculated as δ = *θπ*/2. A compact (clot) network structure is reflected by a higher value of D_f_, whereas lower values correspond to more open/permeable networks (Evans et al., 2010).

***Absolute viscosity***: Exactly 7 mL of K EDTA whole blood was injected into a double‐walled concentric rheometer (Discovery Hybrid‐2, TA Instruments) for analysis at 37°C. After loading and incubation blood was exposed to a controlled shear of 225 per second, velocity of 4.37 rad/s, stress of 0.03 Pa and torque of 10.5 µN/m for 60 s. After removal of the first two data points, all remaining 46 sample points were averaged and accepted as the absolute viscosity (η) of the blood at time of sampling.

***Coagulometry***: Standard coagulometry assays were also performed using a Ceveron Alpha (Technoclone GmbH, Vienna, Austria) automated coagulometer with corresponding assays (Technoclone GmbHAustria) to measure activated partial thromboplastin time (aPTT), prothrombin time (PT) and D‐dimer.

### Statistical analysis

Prospective power calculations and sample size estimates were performed using G^∗^ Power, version 3.1, software. Pilot data highlighted effect sizes (η^2^) of 1.410 and 2.100 for A^•−^ and D_f_, respectively, in a comparable group of participants requiring a (minimum) sample size of seven and five participants, respectively, to achieve a power (1–ß) of 0.80 at *P* < 0.05. We chose to inflate this to eight participants given the (albeit unlikely) potential for incomplete data collection. Data were analysed via IBM SPSS Statistics 29.0 (IBM, NY, USA) using a two‐factor mixed ANOVA (group: TSST *vs*. control × state: pre *vs*. post) after confirmation of distribution normality using Shapiro–Wilk W tests (*P* > 0.05). *Post hoc* comparisons were conducted using Bonferroni‐corrected paired samples *t* tests. Significance was established at *P* < 0.05 for all two‐tailed tests, and data are presented as mean ± SD.

## Results

Full data, including calculations of percentage differences (Δ), are presented in Table 1.

**Table 1:** Impact of TSST and control on biomarkers of free radical formation, clot microstructure and coagulation

		D_f_	η	aPTT	PT	D‐dimer	A^•–^
		(AU)	(CP)	(s)	(s)	(ng/mL)	(AU)
**Stress**	**Pre**	1.72 (0.03)	4.27 (0.73)	40.1 (4.1)	18.6 (3.3)	176 (106)	215 (49)
	**Post**	1.81 (0.03)^a^, ^*^	4.57 (0.51)	35.6 (4.5)^a^	15.6 (1.5)	167 (112)	258 (47)^a^, ^*^
	Δ	5% (3%)	9% (13%)	−11% (7%)	−14% (16%)	26% (96%)	23% (19%)
**Control**	**Pre**	1.72 (0.02)	4.19 (0.63)	38.0 (3.7)	16.2 (1.3)	211 (105)	208 (54)
	**Post**	1.73 (0.03)	4.47 (0.76)	36.5 (4.8)	16.7 (1.9)	208 (83)	216 (49)
	**Δ**	0% (2%)	7% (8%)	−4% (6%)	4% (13%)	25% (71%)	5% (15%)

*Note*: Values are means (±SD).

Abbreviations: A^•–^, ascorbate free radical; aPTT, activated partial thromboplastin time; D_f_, fractal dimension; PT, prothrombin time; TSST, Trier Social Stress Test; η, absolute viscosity; Δ, percentage change *versus* baseline.

^a^ Denotes significantly different to pre‐stress.

* Denotes significantly different to post‐control (*P*<0.05).

### Free radicals

**Figure 1:**
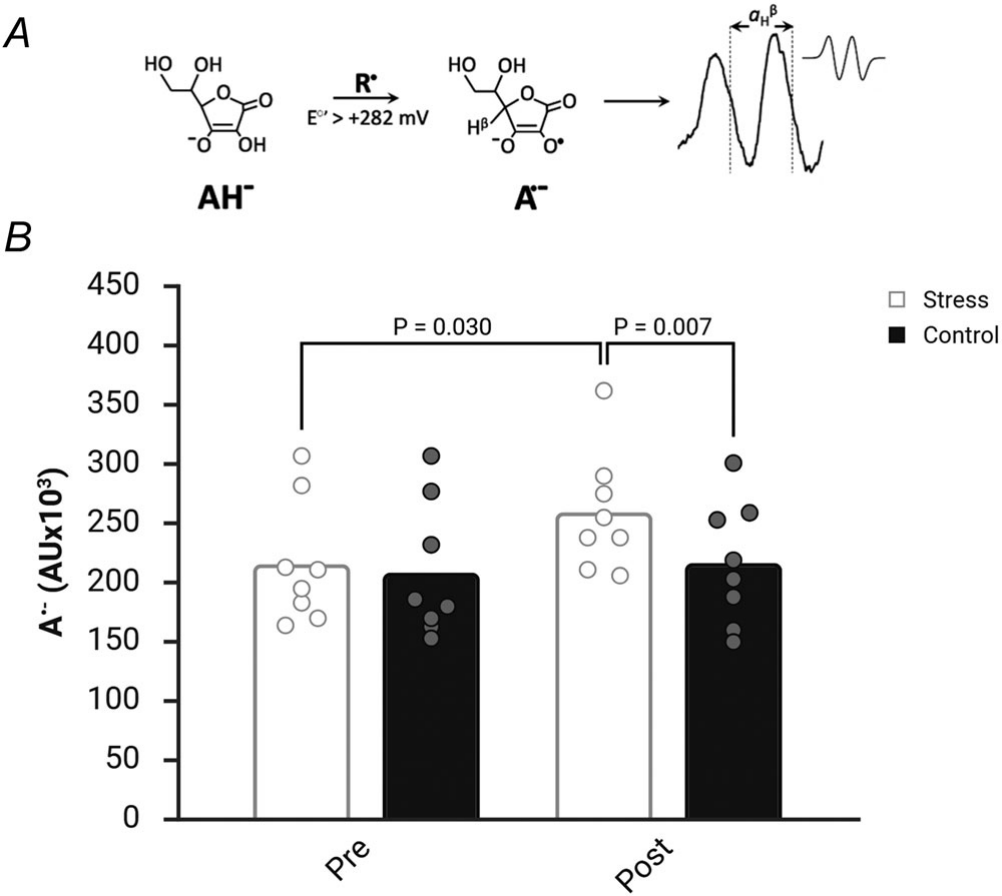
Systemic free radical formation *A*, at the current settings A^•−^ appears as a filtered doublet with a hydrogen hyperfine coupling constant (aHβ) of ∼1.76 G (see top inset for simulated spectrum; Fall et al., 2023). *B*, values are mean ± SD based on *n* = 8. A^•−^, ascorbate radical; AU, arbitrary units. Created in https://BioRender.com.

The general reaction principles underlying A^• −^ formation and the corresponding changes during the protocol are illustrated in Fig. 1*A,B*. There were no basal differences discovered between trials (*P* = 1.000), and unlike the quiet sitting control, the TSST increased A^• −^ (*P* = 0.030 *vs*. pre‐TSST; *P* = 0.070 *vs*. control).

### Rheology

**Figure 2:**
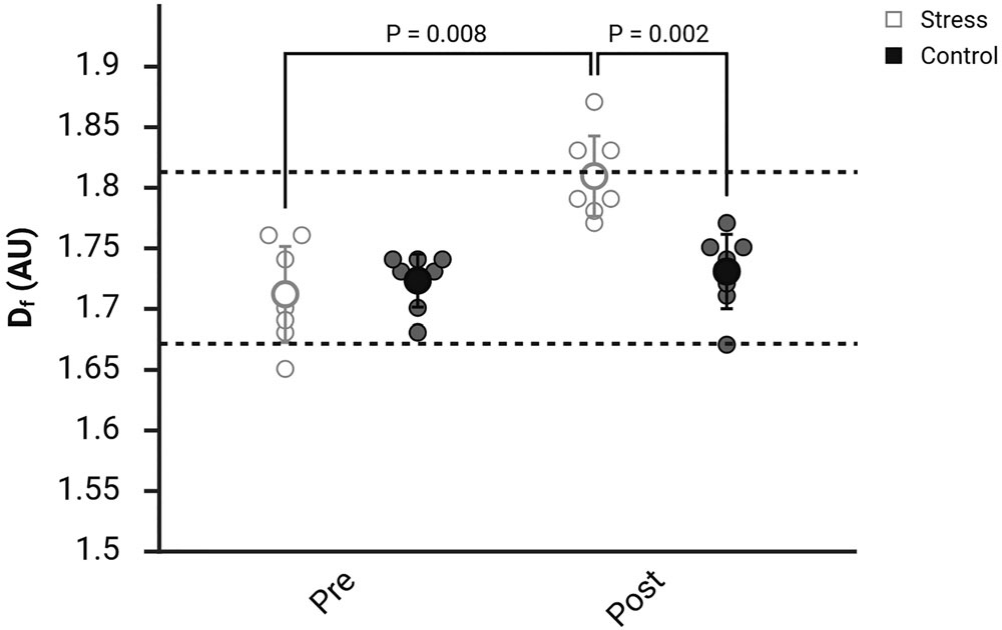
Insipient clot microstructure Values are mean ± SD (*n* = 8). D_f_, fractal dimension; AU, arbitrary units. The dashed lines represent the accepted reference range in healthy humans (Evans et al., 2010); that is, D_f_ = 1.67–1.81 AU. Created in https://BioRender.com.

D_f_: No baseline differences were observed between trials for D_f_ (*P* = 0.655). The TSST increased D_f_ (Fig. 2, *P* = 0.008 *vs*. pre‐TSST; *P* = 0.002 *vs*. control). η exhibited no basal differences and remained unchanged after both the TSST and quiet sitting (*P* = 1.000 pre‐/post‐TSST, *P* = 0.2706 pre‐/post‐control).

**Figure 3:**
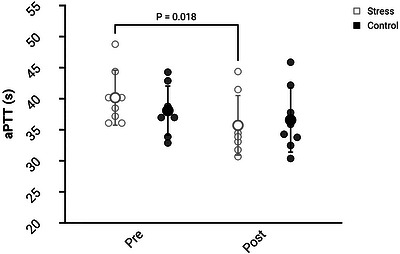
Intrinsic coagulation pathway activation Values are mean ± SD (*n* = 8). aPTT, activated partial thromboplastin time. Created in https://BioRender.com.

***aPTT***: No baseline differences were observed in aPTT (Fig. 3). The TSST shortened aPTT (*P* = 0.002 *vs*. pre‐TSST), whereas quiet sitting produced no change (*P* = 0.818 *vs*. pre‐TSST).

***PT***: No differences were observed at baseline (*P* = 0.4959), after the TSST (*P* = 0.296 *vs*. pre‐TSST) or quiet sitting (*P* = 1.000 *vs*. pre‐control).

***D‐dimer***: No differences were observed between interventions or timepoints (*P* = 1.000 in all cases).

## Discussion

The present findings provide unique insight into the basic mechanisms underlying the redox regulation of stress‐induced activated coagulation. First, acute psychological stress increased systemic free radical formation, evidenced by elevated A^•−^, accompanied by corresponding alterations in incipient blood clot microstructure reflected by an increased D_f_. Second, these changes were associated with shortened activation of the intrinsic coagulation pathway, indicated by reduced aPTT, with no changes in PT or D‐dimer. Third these effects occurred without alterations in blood viscosity. Taken together these findings extend current understanding of the haemostatic stress response by providing the first *in vivo* evidence that oxidative stress serves as a primary mechanistic driver through which acute psychological distress triggers prothrombotic structural remodelling of the fibrin network.

### Psychological stress–induced free radical formation

We specifically employed EPR spectroscopy to detect A^•–^ as a direct biomarker of global free radical formation. This approach, which we have applied previously to investigate free radical–mediated activation of coagulation, offers advantages in experimental control and interpretability compared with approaches targeting specific radical species using complex *ex vivo* spin‐trapping techniques (Villamena & Zweier, 2004).

Because the concentration of ascorbate in human plasma is orders of magnitude greater than that of any oxidising species, and the one‐electron reduction potential of the A•^−^/ascorbate monoanion (AH^−^) couple is relatively low (E°′ ≈ 282 mV) (Williams & Yandell, 1982), oxidising processes within the circulation readily drive the one‐electron oxidation of ascorbate. Importantly, this reaction is not limited to direct interaction with free radical species (R•); ascorbate can also undergo one‐electron oxidation via redox cycling with transition metals such as Fe^3+^, Cu^2+^ or haeme‐associated iron. Consequently elevations in A^•–^ may reflect both radical‐mediated oxidation and increased availability or catalytic cycling of redox‐active metal species within the systemic circulation (Bailey et al., 2006, 2014, 2022). The resulting ascorbate radical is resonance stabilised because the unpaired electron is delocalised across a conjugated tri‐carbonyl π‐system, permitting direct detection of the characteristic A^•–^ doublet using EPR spectroscopy (R• + AH^−^ → A•^−^ + R–H) (Buettner, 1993). Because the steady‐state concentration of A^•−^ is governed by well‐defined redox thermodynamics and reflects the dynamic balance between oxidant generation, metal‐catalysed redox cycling and antioxidant capacity, its direct detection provides an integrative indicator of systemic oxidant load rather than a narrow biomarker of free radical formation alone.

That the TSST, but not the quiet sitting control, elicited a significant elevation in A^•–^ provides strong evidence that acute psychological stress increases systemic free radical formation. Prolonged stress is known to induce mitochondrial dysfunction and increase reactive oxygen species (ROS) production (Duclos et al., 2004; Orzechowski et al., 2002), but our findings provide direct *in vivo* evidence that this response also occurs after acute psychological stress in humans. This contrasts with suggestions that acute stress may instead stimulate mitochondrial biogenesis and increased respiratory chain enzymatic activity (Manoli et al., 2007). Recent work using the TSST reported no increase in oxidative stress (Janšáková et al., 2021); however that study relied on saliva‐based, indirect biomarkers of lipid peroxidation and exhibited elevated baseline values prior to the TSST relative to control conditions, potentially reflecting a previously described free radical ‘ceiling effect’ (Fall et al., 2023). The present study is the first to apply EPR spectroscopy to directly quantify systemic free radical formation during acute psychological stress, providing a more controlled mechanistic assessment. Coupled with our previous demonstrations of a causal relationship between oxidative stress and coagulation activation (Fall et al., 2015, 2018, 2023), the concurrent increase in D_f_ and shortening of aPTT strongly support oxidative stress as a primary mechanistic driver of stress‐induced coagulation activation.

The precise biological link between emotional stress and haemostatic activation lies beyond the scope of this initial study. However we hypothesise that activation of the HPA axis activation, a well‐established response to the TSST (Kudielka et al., 2004), particularly in males (Uhart et al., 2006), drives cortisol release from the adrenal cortex via adrenocorticotropic hormone (Choi & Han, 2021). Through glucocorticoid receptor‐Bcl‐2 signalling this may promote translocation of the anti‐apoptotic Bcl‐2 protein to the mitochondria (Du, McEwen et al., 2009), increasing mitochondrial membrane potential, calcium‐holding capacity and oxidative metabolism. The resulting elevation in cellular metabolic rate enhances ATP production but also promotes spontaneous superoxide (O_2_•^−^) generation at complexes I and III of the electron transport chain (Choi & Han, 2021). This is however a hypothesis that requires formal experimental confirmation.

### Rheology

In the present study Fourier transformation haemorheology was used to derive our primary biomarker of activated coagulation, D_f_. In contrast to quiet sitting the TSST elicited a significant elevation in D_f_. In healthy individuals D_f_ typically averages 1.74 ± 0.04 (Evans et al., 2010); thus the increases observed in our participants exceed the normal (i.e. healthy) reference range. This indicates the formation of larger, thicker and more fibrin‐dense clots (Badiei et al., 2015), a more compact and less‐permeable incipient clot microstructure consistent with a prothrombotic shift in clot architecture after acute psychological stress.

This increase in D_f_ occurred without concomitant changes in η, indicating no alteration in bulk blood viscosity and supporting our previous criticism of the haemoconcentration hypothesis (Fall & Bailey, 2013). Instead the findings suggest that oxidative stress may act upstream to modify fibrin network architecture directly, producing a denser, more compact clot microstructure. Thus the TSST appears to influence clot quality rather than bulk rheological properties or the initial kinetics of polymerisation, underscoring the sensitivity of D_f_ as a measure of clot microstructure.

To the best of our knowledge, this is the first study to examine clot microstructural responses to acute psychological stress, limiting direct comparison with prior work. However if increased D_f_ reflects a prothrombotic phenotype, the present findings provide rheological support for biochemical evidence that psychological stress promotes thrombogenicity (Hamer et al., 2006; Larsson et al., 1989; Rosovsky et al., 2024; Steptoe & Marmot, 2006; Wirtz et al., 2006, 2009).

### Traditional biomarkers of coagulation

Alongside the haemorheological measurements we assessed conventional coagulation biomarkers and observed a significant shortening of aPTT after the TSST, whereas quiet sitting elicited no changes. These findings suggest that acute psychological stress activates the intrinsic/contact pathway of coagulation, consistent with previous reports demonstrating aPTT shortening after acute stress (Austin et al., 2012; Von Känel et al., 2009). In contrast PT and D‐dimer remained unchanged, indicating that the degree of intrinsic pathway activation was insufficient to elicit measurable fibrinolysis. This pattern aligns with the TSST representing a subclinical stressor, although it contrasts with earlier studies reporting stress‐related increases in D‐dimer (Austin et al., 2013; Von Känel, 2015; von Känel et al., 2001, 2002, 2004; Wirtz et al., 2006). Collectively these data indicate that the TSST modulated haemostasis, primarily through activation of the intrinsic/contact coagulation pathway.

### Limitations and future directions

Several limitations warrant consideration. First although the sample size was small (*n* = 8), the observed statistical power for the primary outcome (D_f_) was 0.85; nevertheless larger studies are required to confirm these preliminary findings. Second the cohort comprised only young, healthy biological males, limiting generalisability to females, older adults or individuals with comorbidities. This design was intentional, as the study aimed to isolate mechanisms linked to HPA axis activation, which is known to exhibit greater stress responsiveness in males. Restricting recruitment therefore maximised experimental signal relative to biological variability. Future studies should extend these findings to biological females and more diverse clinical populations.

### Conclusion

This study provides the first *in vivo* evidence that acute psychological stress increases systemic free radical formation, accompanied by prothrombotic alterations in clot microstructure. TSST‐induced elevations in A^•−^ and D_f_, together with shortened aPTT, indicate free radical–mediated activation of the intrinsic coagulation pathway. Collectively these findings demonstrate that even brief emotional stress can modify clot architecture and increase thrombotic potential, identifying oxidative stress as a key proximal mechanistic link, and a potential therapeutic target, in stress‐related CVD.

## Additional information

## Competing interests

Damian M. Bailey is editor‐in‐chief of *Experimental Physiology*, chair of the Life Sciences Working Group, member of the Human Spaceflight and Exploration Science Advisory Committee to the European Space Agency, member of the Space Exploration Advisory Committees to the UK and Swedish National Space Agencies, member of the National Cardiovascular Network for Wales and South‐East Wales Vascular Network and founding member of The International Academic Surgical Consortium (THALAMUS).

## Author contributions

L.F. and D.M.B. conceived the idea and wrote the first draft of the manuscript. All authors edited and revised the manuscript. All authors approved the final version submitted for publication and agree to be accountable for all aspects of the work in ensuring that questions related to the accuracy or integrity of any part of the work are appropriately investigated and resolved. All persons designated as authors qualify for authorship, and all those who qualify for authorship are listed.

## Funding

D.M.B. is supported by a Royal Society Wolfson Research Fellowship (grant no. WM170007).

## Supporting information


Peer Review History

## Data Availability

Data are available from the corresponding author upon reasonable request and approval of a data‐sharing agreement.
