# Supplementary material for: Psychological stress catalyses free radical–mediated activation of coagulation in humans
Source: J Physiol. 2026 Apr 25;604(12):4707–16. doi: 10.1113/JP290576 (PMC13267672; doi:10.1113/JP290576)
Supplement: Supplementary file 1 — Peer Review History [file TJP-604-4707-s001.pdf]

The Journal of Physiology

Psychological stress catalyses free radical-mediated activation of coagulation in humans

Lewis Fall, Benjamin S Stacey, Elsie Swain, Ebony Samuel, Meghan Robinson, Danny W Walmsley, Christopher Marley, Thomas Simon Owens, Adnan Haq, Leon Garfield Yandle, Kevin George Cox, and Damian Miles Bailey
DOI: 10.1113/JP290576

Corresponding author(s): Lewis Fall (lewis.fall@southwales.ac.uk)

The following individual(s) involved in review of this submission have agreed to reveal their identity: Eric E Kelley (Referee #1)

Review Timeline:

Submission Date:	20-Nov-2025
Editorial Decision:	06-Jan-2026
Revision Received:	10-Mar-2026
Editorial Decision:	31-Mar-2026
Revision Received:	01-Apr-2026
Accepted:	08-Apr-2026

Senior Editor: Bjorn Knollmann

Reviewing Editor: Joseph Welch

Transaction Report:

(Note: With the exception of the correction of typographical or spelling errors that could be a source of ambiguity, letters and reports are not edited. Depending on transfer agreements, referee reports obtained elsewhere may or may not be included in this compilation. Referee reports are anonymous unless the Referee chooses to sign their reports.)

Re: JP-RP-2025-290576 "**Psychological stress catalyses free radical-mediated activation of coagulation in humans**" by Lewis Fall, Benjamin S Stacey, Elsie Swain, Ebony Samuel, Meghan Robinson, Danny Walmsley, Christopher Marley, Thomas Simon Owens, Adnan Haq, Leon Garfield Yandle, Kevin Cox, and Damian Miles Bailey

Dear Dr Fall,

Thank you for submitting your manuscript to The Journal of Physiology. It has been assessed by a Reviewing Editor and by 1 expert referees and we are pleased to tell you that it is potentially acceptable for publication following satisfactory major revision.

Please advise your co-authors of this decision as soon as possible.

The referee reports are copied at the end of this email.

Please address all the points raised and incorporate all requested revisions or explain in your Response to Referees why a change has not been made. We hope you will find the comments helpful and that you will be able to return your revised manuscript within 2 months. If your article is NOT for a Special Issue, you may have 9 months to revise. If you require an extension, please contact journal staff: jp@physoc.org. Please note that this letter does not constitute a guarantee for acceptance of your revised manuscript.

Your revised manuscript should be submitted online using the link in your Author Tasks: Link Not Available. This link is accessible via your account as Corresponding Author; it is not available to your co-authors. If this presents a problem, please contact journal staff (jp@physoc.org). Image files from the previous version are retained on the system. Please ensure you replace or remove any files that are being revised.

If you do not wish to submit a revised version of your manuscript, you must inform our journal staff (jp@physoc.org) or reply to this email to request withdrawal. Please note that a manuscript must be formally withdrawn from the peer review process at one journal before it may be submitted to another journal.

TRANSPARENT PEER REVIEW POLICY: To improve the transparency of its peer review process, The Journal of Physiology publishes online as supporting information the peer review history of all articles accepted for publication. Readers will have access to decision letters, including Editors' comments and referee reports, for each version of the manuscript, as well as any author responses to peer review comments. Referees can decide whether or not they wish to be named on the peer review history document.

ABSTRACT FIGURES: Authors are expected to use The Journal's premium BioRender account to create/redraw their Abstract Figures. Information on how to access this account is here: https://physoc.onlinelibrary.wiley.com/journal/14697793/biorender-access.

This will enable Authors to create and download high-resolution figures. If authors have used the free BioRender service, they can use the instructions provided in the link above to download a high-resolution version suitable for publication.

The link provided should only be used for the purposes of this submission. Authors will be charged for figures created on this account if they are not related to this manuscript submission.

LANGUAGE EDITING AND SUPPORT FOR PUBLICATION: If you would like help with English language editing, or other article preparation support, Wiley Editing Services offers expert help, including English Language Editing, as well as translation, manuscript formatting, and figure formatting at www.wileyauthors.com/eeo/preparation. You can also find resources for Preparing Your Article for general guidance about writing and preparing your manuscript at www.wileyauthors.com/eeo/prepresources.

RIGOUR AND REPRODUCIBILITY: We recommend authors consult The Journal's Rigour and Reproducibility requirements to ensure that their manuscript is suitable for publication: https://physoc.onlinelibrary.wiley.com/pb-assets/hub-assets/physoc/documents/TJP-Rigour-and-Reproducibility-Requirements.pdf

REVISION CHECKLIST:

Check that your Methods section conforms to journal policy: https://jp.msubmit.net/cgi-bin/main.plex?form_type=display_requirements#methods.

Check that data presented conforms to the statistics policy: https://jp.msubmit.net/cgi-bin/main.plex?form_type=display_requirements#statistics.

Upload a full Response to Referees file. To create your 'Response to Referees': copy all the reports, including any comments from the Senior and Reviewing Editors, into a Microsoft Word, or similar, file and respond to each point, using font or background colour to distinguish comments and responses and upload as the required file type.

Please upload two versions of your manuscript text: one with all relevant changes highlighted and one clean version with no changes tracked. The manuscript file should include all tables and figure legends, but each figure/graph should be uploaded as separate, high-resolution files.

You may also upload:

- 'Potential Cover Art' for consideration as the issue's cover image
- Appropriate Supporting Information (Video, audio or data set: see https://jp.msubmit.net/cgi-bin/main.plex?form_type=display_requirements#supp).

We look forward to receiving your revised submission.

If you have any queries, please reply to this email and we will be pleased to advise.

Yours sincerely,

Bjorn Knollmann
Senior Editor
The Journal of Physiology

REQUIRED ITEMS

1) - Include a Key Points list in the article itself, before the Abstract.

2) - Author photo and profile. First or joint first authors are asked to provide a short biography (no more than 100 words for one author or 150 words in total for joint first authors) and a portrait photograph. These should be uploaded and clearly labelled together in a Word document with the revised version of the manuscript. See Information for Authors for further details.

3) - You must start the Methods section with a paragraph headed Ethical Approval. If experiments were conducted on humans, confirmation that informed consent was obtained, preferably in writing, that the studies conformed to the standards set by the latest revision of the Declaration of Helsinki and that the procedures were approved by a properly constituted ethics committee, which should be named, must be included in the article file. If the research study was registered (clause 35 of the Declaration of Helsinki), the registration database should be indicated, otherwise the lack of registration should be noted as an exception (e.g. The study conformed to the standards set by the Declaration of Helsinki, except for registration in a database). For further information see: https://physoc.onlinelibrary.wiley.com/hub/human-experiments.

4) - The reference list must be in alphabetical order, rather than numbered, to comply with our Journal format.

5) - A Data Availability Statement is required for all papers reporting original data. This must be in the Additional Information section of the manuscript itself. It must have the paragraph heading 'Data Availability Statement'. All data supporting the results in the paper must be either: in the paper itself; uploaded as Supporting Information for Online Publication; or archived in an appropriate public repository. The statement needs to describe the availability or the absence of shared data. Authors must include in their statement: a link to the repository they have used, or a statement that it is available as Supporting Information; reference the data in the appropriate sections(s) of their manuscript; and cite the data they have shared in the References section. Whenever possible, the scripts and other artefacts used to generate the analyses presented in the paper should also be publicly archived. If sharing data compromises ethical standards or legal requirements then authors are not expected to share it, but must note this in their statement. For more information, see our Statistics Policy.

6) - Please include an Abstract Figure file and an Abstract Figure legend. An appropriate figure legend, which should not exceed 150 words in length, should be included in the main manuscript file. The Abstract Figure is a piece of artwork designed to give readers an immediate understanding of the research and should summarise the main conclusions. If possible, the image should be easily 'readable' from left to right or top to bottom. It should show the physiological relevance of the manuscript so readers can assess the importance and content of its findings. Abstract Figures should not merely

recapitulate other figures in the manuscript. Please try to keep the diagram as simple as possible and without superfluous information that may distract from the main conclusion(s). Abstract Figures must be provided by authors no later than the revised manuscript stage and should be uploaded as a separate file during online submission labelled as File Type 'Abstract Figure'. Please also ensure that you include the figure legend in the main article file. All Abstract Figures should be created using BioRender. Authors should use The Journal's premium BioRender account to export high-resolution images. Details on how to use and access the premium account are included as part of this email.

7) - Please include a full title page as part of your main article (Word) file, which should contain the following: title, authors, affiliations, corresponding author name and contact details, keywords, and running title.

8) - Please ensure that all figures and tables have a title and legend, and that they have been cited within the main article text.

EDITOR COMMENTS

Reviewing Editor:

The study examined the mechanisms of coagulation induced by psychological stress (Trier Social Stress Test) measured via venous blood sampling and analysis of free radical formation and prothrombotic clot formation using electron paramagnetic resonance spectroscopy and Fourier transform rheology in eight healthy males. The key findings were: 1) increased ascorbate free radical and fractal dimension (an indicator of clot microstructure); and 2) shortened activated partial thromboplastin time. These data suggest that free-radical mediated oxidative stress underpins prothrombotic remodelling of clot microstructure via activation of the intrinsic coagulation pathway. The study appears to be well conducted, and the data are compelling, adding to our understanding of mechanisms of haemostatic activation triggered by emotional stress. However, I have a few concerns:

1. The introduction is short and lacks sufficient detail to fully understand the rationale/purpose of the investigation. Further, no hypothesis is presented. I encourage the authors to expand on the study rationale.

2. The discussion is short and combines the results into one section. No limitations are included. Greater discussion of both the implications of the results and the possible limitations of the study would improve the manuscript.

3. The statistical approach may be improved by comparing relative (%) changes in primary dependent measures in stress and control conditions, rather than just the 2x2 ANOVA on absolute values that is currently presented. This may improve interpretation of findings.

4. The discussion/conclusion may sometimes go beyond the data collected and methods used. For example, whilst increased ascorbate and fractal dimension may point toward upstream clot formation remodelling, it does not unequivocally demonstrate pathway activation. Perhaps the paper could be more cautious about interpretation of mechanism based on these data.

5. Please state if the procedures conformed to the standards of the Declaration of Helsinki, and if informed consent was obtained from participants.

Ethics Concerns:
It is not stated if informed consent was obtained, nor if study procedures followed the standards of the Declaration of Helsinki.

Senior Editor:

I concur with the reviewing editor. A responsive revision would require extensive rewriting of the MS to address the concerns raised in the review. Please expand the introduction, provide a separate discussion and limitations section, and edit the figure and figure legends for clarity. Please provide ethics approval.

REFEREE COMMENTS

Referee #1:

This paper examined the impact of psychological stress on systemic free radical generation by assessing the extent of circulating ascorbate radical using EPR. This technique has been used previously in vivo as a surrogate for "oxidative stress" in rodents, pigs and dogs. However, I believe the technique is underutilized and thus this paper is of value to the field and timely! I have a few comments that would add to the strength of this work as seen below:

1) Please provide greater detail regarding what the appearance of the ascorbate radical means. It is not just an indicator of "free radical" generation via reaction with radical species. It also reacts with metals (Cu, Fe, hemin etc.) to generate a one electron process to generate the ascorbate radical. As such, an alternative "elevation in free radical levels" could be an elevation in redox active iron/heme. Thus, the authors need to give a little more background on redox biochemistry of ascorbate and let the reader know why it is a good indicator of systemic oxidant load. This would be very informative. Papers by Garry Buettner provide a great background for this approach. And, no I am not Buettner looking for a reference.

2) Please refer to EPR as electron paramagnetic spectrometry and not "spectroscopy". EPR does not examine light.

3) In the methods section, please identify the EPR instrument that was used.

END OF COMMENTS

Response to Editors and Referee

We extend our sincere thanks to the Reviewing Editor, Senior Editor and referee for taking the time to review our manuscript. We appreciate their specialist input and helpful critique. Our response provides a brief response to each of the queries raised, including modifications to the revised (R1) text highlighted in red.

EDITOR COMMENTS

Reviewing Editor:

The study examined the mechanisms of coagulation induced by psychological stress (Trier Social Stress Test) measured via venous blood sampling and analysis of free radical formation and prothrombotic clot formation using electron paramagnetic resonance spectroscopy and Fourier transform rheology in eight healthy males. The key findings were: 1) increased ascorbate free radical and fractal dimension (an indicator of clot microstructure); and 2) shortened activated partial thromboplastin time. These data suggest that free-radical mediated oxidative stress underpins prothrombotic remodelling of clot microstructure via activation of the intrinsic coagulation pathway. The study appears to be well conducted, and the data are compelling, adding to our understanding of mechanisms of haemostatic activation triggered by emotional stress. However, I have a few concerns:

1. The introduction is short and lacks sufficient detail to fully understand the rationale/purpose of the investigation. Further, no hypothesis is presented. I encourage the authors to expand on the study rationale.

We thank the reviewer for these helpful comments. The Introduction has since been substantially revised to provide clearer scientific context, expand upon the rationale and significance of the investigation, and better articulate the knowledge gap being addressed. In addition, a clear, testable hypothesis has now been incorporated into the Introduction, as kindly requested.

2. The discussion is short and combines the results into one section. No limitations are included. Greater discussion of both the implications of the results and the possible limitations of the study would improve the manuscript.

We thank the reviewer for this valuable suggestion. The Discussion has now been substantially expanded and restructured to allow clearer interpretation of the key findings. Greater emphasis has been placed on the physiological and translational implications of the results, and a dedicated subsection outlining the study limitations has now been included.

3. The statistical approach may be improved by comparing relative (%) changes in primary dependent measures in stress and control conditions, rather than just the 2x2 ANOVA on absolute values that is currently presented. This may improve interpretation of findings.

We thank the reviewer for this suggestion. The primary statistical approach (2×2 ANOVA on absolute values) has been retained, as this was defined *a priori* and remains central to the interpretation of the data. However, to aid clarity and provide complementary insight, we have additionally examined relative (%) changes in the primary dependent variables between stress and control conditions. These data are now presented as supportive analyses, and the corresponding text has been updated to reflect this with the addition of a table.

4. The discussion/conclusion may sometimes go beyond the data collected and methods used. For example, whilst increased ascorbate and fractal dimension may point toward upstream clot formation remodelling, it does not unequivocally demonstrate pathway activation. Perhaps the paper could be more cautious about interpretation of mechanism based on these data.
We thank the reviewer for this important conceptual point. We agree that the present data do not provide direct evidence of haemostatic pathway activation. Accordingly, the discussion and conclusion have been revised to adopt more cautious language, clearly distinguishing between associative findings and mechanistic inference. References to potential upstream processes are now framed as hypotheses rather than definitive mechanisms, and the text has been tempered to emphasise the exploratory nature of these interpretations.

5. Please state if the procedures conformed to the standards of the Declaration of Helsinki, and if informed consent was obtained from participants.
We apologise for this omission in the original submission. All experimental procedures were conducted in accordance with the principles outlined in the Declaration of Helsinki, with the exception of registration in a public database. Written informed consent was obtained from all participants prior to inclusion in the study. This information has now been added to the Ethical Approval and Participants section of the manuscript.

Ethics Concerns:
It is not stated if informed consent was obtained, nor if study procedures followed the standards of the Declaration of Helsinki.
We thank the reviewer for highlighting this omission. As outlined above, we have now provided a full and explicit clarification.

Senior Editor:
I concur with the reviewing editor. A responsive revision would require extensive rewriting of the MS to address the concerns raised in the review. Please expand the introduction, provide a separate discussion and limitations section, and edit the figure and figure legends for clarity. Please provide ethics approval.
We thank the Senior Editor for this clear and constructive guidance. In response and as outlined in our response herein, we have undertaken a substantial revision of the manuscript. The Introduction has been expanded to provide greater contextual background and to clarify the study rationale and objectives. The Results and Discussion have now been presented as separate sections, and a dedicated Limitations subsection has been added to more appropriately frame a more tempered interpretation of the findings. Figures and corresponding legends have been revised to improve clarity and readability.

With respect to ethics, full details of ethical approval and informed consent have now been explicitly stated in the Ethical Approval and Participants section, confirming that all procedures conformed to the principles of the Declaration of Helsinki.

We hope that these revisions satisfactorily address the concerns raised and improve the overall clarity and rigor of the manuscript.

REFEREE COMMENTS
Referee #1:
This paper examined the impact of psychological stress on systemic free radical generation by assessing the extent of circulating ascorbate radical using EPR. This technique has been used previously in vivo as a surrogate for "oxidative stress" in rodents, pigs and dogs. However, I believe the technique is underutilized and thus this paper is of value to the field and timely! I have a few comments that would add to the strength of this work as seen below:
We thank the referee for these thoughtful and constructive comments and we are particularly grateful for their recognition of the value of the analytical approaches employed. The combined application of EPR spectroscopy with fractal dimension analysis represents the most direct analytical methods currently available *sine qua non* for assessing systemic redox-mediated haemostatic responses in vivo. We have carefully considered the reviewer's suggestions and revised the manuscript accordingly to strengthen clarity and interpretation.

1) Please provide greater detail regarding what the appearance of the ascorbate radical means. It is not just an indicator of "free radical" generation via reaction with radical species. It also reacts with metals (Cu, Fe, hemin etc.) to generate a one electron process to generate the ascorbate radical. As such, an alternative "elevation in free radical levels" could be an elevation in redox active iron/heme. Thus, the authors need to give a little more background on redox biochemistry of ascorbate and let the reader know why it is a good indicator of systemic oxidant load. This would be very informative. Papers by Garry Buettner provide a great background for this approach. And, no I am not Buettner looking for a reference.
We thank the reviewer for this important comment. Accordingly, we have revised the manuscript to provide a more mechanistically grounded interpretation of $A^{\bullet -}$ formation, explicitly framed within the thermodynamics of the one-electron ascorbate/ascorbate monoanion redox couple. We now clarify that elevations in $A^{\bullet -}$ may therefore reflect increased bioavailability or cycling of redox-active iron or haem species, in addition to radical-mediated oxidation. Since $A^{\bullet -}$ formation is governed by well-defined reduction potentials, its steady-state concentration provides an integrative index of systemic oxidant load, capturing the balance between oxidant generation, metal-catalysed redox cycling, and antioxidant capacity, rather than serving as a narrow biomarker of free radical generation, referencing both foundational work by Dr Buettner and our own recent work (PMID: 35398201), albeit in a different clinical context.

2) Please refer to EPR as electron paramagnetic spectrometry and not "spectroscopy". EPR does not examine light.
We thank the reviewer for raising this point and for the opportunity to clarify our use of terminology. We appreciate that spectrometry refers to the direct quantitative measurement of a spectrum (e.g., mass-to-charge ratios in mass spectrometry). While EPR experiments are performed using a spectrometer (the instrument), the discipline itself is spectroscopic in nature, as it interprets resonance phenomena arising from radiation–matter interactions rather than measuring mass or particle flux. This distinction is consistent with long-standing convention across physics, chemistry, and the biomedical sciences. Accordingly, in the revised manuscript we refer to the technique as EPR 'spectroscopy', while correctly identifying the

instrument as an EPR ‘spectrometer’, consistent with our prior published research, ensuring both terminological accuracy and clarity.

3) In the methods section, please identify the EPR instrument that was used.
Thank you for this comment, this has been amended accordingly.

Dear Dr Fall,

Re: JP-RP-2026-290576R1 "**Psychological stress catalyses free radical-mediated activation of coagulation in humans**" by Lewis Fall, Benjamin S Stacey, Elsie Swain, Ebony Samuel, Meghan Robinson, Danny W Walmsley, Christopher Marley, Thomas Simon Owens, Adnan Haq, Leon Garfield Yandle, Kevin Cox, and Damian Miles Bailey

Thank you for submitting your manuscript to The Journal of Physiology. It has been assessed by a Reviewing Editor and by 1 expert referees and we are pleased to tell you that it is acceptable for publication following satisfactory revision.

The referee reports are copied at the end of this email.

Please address all the points raised and incorporate all requested revisions or explain in your Response to Referees why a change has not been made. We hope you will find the comments helpful and that you will be able to return your revised manuscript within 4 weeks. If you require longer than this, please contact journal staff: jp@physoc.org.

Your revised manuscript should be submitted online using the link in your Author Tasks Link Not Available. This link is accessible via your account as Corresponding Author; it is not available to your co-authors. If this presents a problem, please contact journal staff (jp@physoc.org). Image files from the previous version are retained on the system. Please ensure you replace or remove any files that are being revised.

If you do not wish to submit a revised version of your manuscript, you must inform our journal staff (jp@physoc.org) or reply to this email to request withdrawal. Please note that a manuscript must be formally withdrawn from the peer review process at one journal before it may be submitted to another journal.

TRANSPARENT PEER REVIEW POLICY: To improve the transparency of its peer review process The Journal of Physiology publishes online, as supporting information, the peer review history of all articles accepted for publication. Readers will have access to decision letters, including Editors' comments and referee reports, for each version of the manuscript, as well as any author responses to peer review comments. Referees can decide whether or not they wish to be named on the peer review history document.

ABSTRACT FIGURES: Authors are expected to use The Journal's premium BioRender account to create/redraw their Abstract Figures. Information on how to access this account is here: https://physoc.onlinelibrary.wiley.com/journal/14697793/biorender-access.

This will enable Authors to create and download high-resolution figures. If authors have used the free BioRender service, they can use the instructions provided in the link above to download a high-resolution version suitable for publication.

The link provided should only be used for the purposes of this submission. Authors will be charged for figures created on this account if they are not related to this manuscript submission.

LANGUAGE EDITING AND SUPPORT FOR PUBLICATION: If you would like help with English language editing, or other article preparation support, Wiley Editing Services offers expert help, including English Language Editing, as well as translation, manuscript formatting, and figure formatting at www.wileyauthors.com/eeo/preparation. You can also find resources for Preparing Your Article for general guidance about writing and preparing your manuscript at www.wileyauthors.com/eeo/prepresources.

RIGOUR AND REPRODUCIBILITY: We recommend authors consult The Journal's Rigour and Reproducibility requirements to ensure that their manuscript is suitable for publication: https://physoc.onlinelibrary.wiley.com/pb-assets/hub-assets/physoc/documents/TJP-Rigour-and-Reproducibility-Requirements.pdf

REVISION CHECKLIST:

Check that your Methods section conforms to journal policy: https://jp.msubmit.net/cgi-bin/main.plex?form_type=display_requirements#methods

Check that data presented conforms to the statistics policy: https://jp.msubmit.net/cgi-bin/main.plex?form_type=display_requirements#statistics

Upload a full Response to Referees file. To create your 'Response to Referees' copy all the reports, including any comments from the Senior and Reviewing Editors, into a Microsoft Word, or similar, file and respond to each point, using font or background colour to distinguish comments and responses and upload as the required file type.

Please upload two versions of your manuscript text: one with all relevant changes highlighted and one clean version with no changes tracked. The manuscript file should include all tables and figure legends, but each figure/graph should be uploaded as separate, high-resolution files. The journal is now integrated with Wiley's Image Checking service. For further details, see: https://www.wiley.com/en-us/network/publishing/research-publishing/trending-stories/upholding-image-integrity-wileys-

image-screening-service

You may also upload:

- 'Potential Cover Art' for consideration as the issue's cover image
- Appropriate Supporting Information (Video, audio or data set: see https://jp.msubmit.net/cgi-bin/main.plex?form_type=display_requirements#supp).

We look forward to receiving your revised submission.

If you have any queries, please reply to this email and we will be pleased to advise.

Yours sincerely,

Bjorn Knollmann
Senior Editor
The Journal of Physiology

EDITOR COMMENTS

Reviewing Editor:

Thank you for addressing the comments/concerns previously raised. I have a few final minor comments:

1. Consider adding a key point to the Key Points Summary about the study rationale/primary research question.

2. L95: I recommend merging the paragraph starting 'The literature further suggests...' with the prior paragraph.

3. Please ensure all statistical results, including P-values, are written to three decimal points.

4. Please ensure all abbreviations are absolutely necessary.

5. L221 and elsewhere: Please report the full P-value, not P>0.05.

6. L239: It might be worth adding a brief sentence to interpret these main findings.

7. L294: I recommend concluding this paragraph by restating that this is a hypothesis and requires experimental confirmation.

Senior Editor:

I concur with the reviewing editor. The work is potentially acceptable after addressing the remaining issues raised by the reviewing editor.

REFEREE COMMENTS

Referee #1:

The authors have addressed my concerns.

END OF COMMENTS

EDITOR COMMENTS

Reviewing Editor:

Thank you for addressing the comments/concerns previously raised. I have a few final minor comments:

We thank the reviewers and editor for taking the time to review the article and the helpful feedback given. We have addressed all points below in green and include a tracked changes version of the manuscript and clean version for consideration.

1. Consider adding a key point to the Key Points Summary about the study rationale/primary research question.

We thank the reviewer for this suggestion. In response, we have merged the previous key points 1 and 2 into a single point and included a new key point 1 of rationale.

2. L95: I recommend merging the paragraph starting 'The literature further suggests...' with the prior paragraph.

We thank the reviewer for this suggestion and have merged the paragraphs.

3. Please ensure all statistical results, including P-values, are written to three decimal points.

Done.

4. Please ensure all abbreviations are absolutely necessary.

We believe all abbreviations are necessary

5. L221 and elsewhere: Please report the full P-value, not P>0.05.

Done

6. L239: It might be worth adding a brief sentence to interpret these main findings.

We thank the reviewer for pointing this out. We have inserted this sentence after the first paragraph of the discussion.

7. L294: I recommend concluding this paragraph by restating that this is a hypothesis and requires experimental confirmation.

We thank the reviewer for pointing this out and have done just that.

Senior Editor:

I concur with the reviewing editor. The work is potentially acceptable after addressing the remaining issues raised by the reviewing editor.

REFEREE COMMENTS

Referee #1:

The authors have addressed my concerns.

Dear Dr Fall,

Re: JP-RP-2026-290576R2 "**Psychological stress catalyses free radical-mediated activation of coagulation in humans**" by Lewis Fall, Benjamin S Stacey, Elsie Swain, Ebony Samuel, Meghan Robinson, Danny W Walmsley, Christopher Marley, Thomas Simon Owens, Adnan Haq, Leon Garfield Yandle, Kevin George Cox, and Damian Miles Bailey

We are pleased to tell you that your paper has been accepted for publication in The Journal of Physiology.

The last Word (or similar) version of the manuscript provided will be used by the Production Editor to prepare your proof. When this is ready you will receive an email containing a link to Wiley's Online Proofing System. The proof should be thoroughly checked and corrected as promptly as possible.

Authors should note that it is too late at this point to offer corrections prior to proofreading. Major corrections at proof stage, such as changes to figures, will be referred to the Editors for approval before they can be incorporated. Only minor changes, such as to style and consistency, should be made at proof stage. Changes that need to be made after proof stage will usually require a formal correction notice.

All queries at proof stage should be sent to: TJP@wiley.com.

Following copyediting and typesetting the accepted version will be published online.

Yours sincerely,

Bjorn Knollmann
Senior Editor
The Journal of Physiology

IMPORTANT POINTS TO NOTE FOLLOWING ACCEPTANCE OF YOUR PAPER:

• IMPORTANT NOTICE ABOUT OPEN ACCESS: To assist authors whose funding agencies mandate immediate public access to published research findings, The Journal of Physiology allows authors to pay an Open Access (OA) fee to have their papers made freely available immediately on publication.

The Corresponding Author will receive an email from Wiley with details on how to register or log in to Wiley Authors where you will be able to place an order.

• You can check if your funder or institution has a Wiley Open Access Account here:
https://authors.wiley.com/author-resources/Journal-Authors/open-access/author-compliance-tool.html

• TRANSPARENT PEER REVIEW POLICY: To improve the transparency of its peer review process, The Journal of Physiology publishes online as supporting information the peer review history of all articles accepted for publication. Readers will have access to decision letters, including Editors' comments and referee reports, for each version of the manuscript as well as any author responses to peer review comments. Referees can decide whether or not they wish to be named on the peer review history document.

• You can help your research get the attention it deserves! Check out Wiley's free Promotion Guide for best-practice recommendations for promoting your work at: www.wileyauthors.com/eeo/guide. You can learn more about Wiley Editing Services which offers professional video, design, and writing services to create shareable video abstracts, infographics, conference posters, lay summaries, and research news stories for your research at: www.wileyauthors.com/eeo/promotion.

• If you would like to receive our 'Research Roundup', a monthly newsletter highlighting the cutting-edge research published in The Physiological Society's family of journals (The Journal of Physiology, Experimental Physiology, Physiological Reports, The Journal of Nutritional Physiology and The Journal of Precision Medicine: Health and Disease), please click this link, fill in your name and email address and select 'Research Roundup':
https://www.physoc.org/journals-and-media/membernews

EDITOR COMMENTS

Reviewing Editor:

Thank you for addressing the final few comments.

Senior Editor:

The work is now acceptable for publication. Thank you for your excellent contribution to the Journal!
